# Right ventricular infarction: epidemiological, clinical, and angiographic characteristics and the outcomes through the experience of a Moroccan cardiology department

**DOI:** 10.1097/MS9.0000000000001528

**Published:** 2023-11-28

**Authors:** Youssra Bouhaddoune, Amine Bouchlarhem, Zakaria Bazid, Nabila Ismaili, Noha El Ouafi

**Affiliations:** a Department of Cardiology, Mohammed VI University Hospital of Oujda; b Laboratory of Epidemiology, Clinical Research and Public Health, Faculty of Medicine and Pharmacy, Mohammed the First University of Oujda, Morocco

**Keywords:** acute myocardial infarction, angioplasty, case series, coronary, right ventricle

## Abstract

**Background:**

Acute myocardial infarction (MI) is a major cause of cardiovascular mortality, which is the leading cause of death in the world. Our objective in this study was to evaluate the epidemiological, clinical, and angiographic features of right ventricular infarction (RVI), as well as its complications and its therapeutic approaches.

**Patients and methods:**

It is a single-centered retrospective descriptive study conducted over a period of 2 years from November 2018 to October 2020. The authors included 82 patients with RVI hospitalized in the cardiovascular ICU during the initial phase of acute coronary syndrome with persistent ST segment elevation. Patients who were diagnosed with RVI at electrocardiogram and echocardiography were recruited.

**Results:**

The authors included 500 patients hospitalized for STEMI, 82 had MI extended to the RV, reflecting a rate of 16.4%. The mean age in our study was 64±12.3 years. Dyslipidemia, diabetes mellitus, and hypertension were the most common cardiovascular risk factors among these patients. RVI co-existed with inferior MI in 62.2 of cases and in 37.8% of anterior MI, while isolated RVI was seen in only one patient. Transthoracic echocardiography showed right ventricular (RV) systolic dysfunction in 24.39% of cases, while RV dilatation was seen in only 10.9% of patients. Therapeutic approach was based essentially on revascularization with thrombolysis and coronary angiography +/- PCI. The percentage of mortality was 2.4%.

**Conclusion:**

RVI is relatively rare and is mostly related to an extension of an inferior MI. Early diagnosis, prompt treatment, and appropriate are the keys to improve prognosis, and reduce complications.

## Introduction

Highlights

- Myocardial infarction (MI) can affect any myocardial territory; however, isolated right ventricle infarction is very rare.
- MI is a major cause of morbidity and mortality.
- Early diagnosis of acute right ventricular myocardial infarction is essential to initiate appropriate therapy.
- All patients with inferior wall MI should have right-sided precordial leads.

Coronary heart disease represents a major public health problem worldwide. Myocardial infarction (MI) remains the main cause of morbidity and mortality^1^. Its incidence is rising in developing countries due to the reappearance of cardiovascular risk factors. Right ventricular infarction (RVI) corresponds to a necrosis of its free wall following a proximal occlusion of the right coronary artery or the circumflex artery when it is dominant^2^. Acute right ventricular myocardial infarction (RVMI) is frequently associated with acute inferior wall MI (30–50%) and less frequently with acute anterior wall MI; this association worsens the prognosis with a mortality of about 30% compared to isolated left ventricular infarctions (6%). Right ventricular (RV) involvement often leads to worse clinical outcomes, such as cardiogenic shock and in-hospital mortality. Therefore, a rapid diagnosis and appropriate treatment of RV infarction is extremely important.

The objective of this work is to evaluate the epidemiological, clinical, and angiographic features of RVI, as well as its complications and its therapeutic approaches and compare our findings to those reported in the literature.

## Materials and methods

### Study setting

This study was carried at the Department of Cardiology between November 2018 and October 2020. This hospital is a tertiary referral hospital for the oriental region of North-Eastern Moroco, the hospital provides medical and surgical services to patients. It is used to train students and graduate doctors from moroccan universities and contributes to scientific research.

### Type of the study

To accomplish our research, we conducted a single-center retrospective study in the cardiovascular ICU for the patients admitted between November 2018 and October 2020. Diagnosis of RV infarction was based on the electrocardiogram (ECG) (a significant ST-elevation in V3R-V4R or a ST elevation in V1 associated with a sub-shift in V2), transthoracic echocardiography (disorder of the global or segmental kinetics of RV), and especially coronary angiography (occlusion of the right coronary artery or the circumflex artery when it is dominant). Our sample size was 500 confirmed patients with STEMI admitted to our department, 82 had MI extended to the right ventricle included in our study.

## Study Participations

### Inclusion criteria

Male and female patients of all ages hospitalized in our cardiac department for MI and those with postinfarction beyond 24 h were included in the study, and then patients with electrocardiographic or echocardiographic criteria for RVI were selected (Table 1).

**Table 1:** Distribution of STEMI according to the territory.

Years	Total number of STEMI	Number of Inferior STEMI	Number of Inferior STEMI with RV infarction	Number of anterior STEMI	Number of anterior STEMI with RV infraction
2018	**180**	**132**	**18**	**20**	**10**
2019	**150**	**105**	**13**	**23**	**9**
2020	**170**	**123**	**20**	**15**	**12**
Total	500	**360**	51	**58**	31

Bold values are statistical significance.

### Exclusion criteria

Patients with pulmonary hypertension, myocarditis, chronic lung disease for pulmonale, left bundle-branch block, and those with previous ECG changes consistent with myocardial ischemia but without new evidence of acute coronary disease were excluded from the study.

### Data collection

Data collection was performed according to the United States National Cardiovascular Data Registry (NCDR) Cath-PCI Registry^3^.

The retrospective analysis was done by collecting data from computerized medical records on the electronic information system. For each patient, the collection considered demographic information (sex and age), socio-demographic data (marital status, personal, and family history), characteristics of chest pain (type, intensity, location, and irradiation), dyspnea (according to the NYHA classification), and palpitations. Cardiovascular risk factors have been investigated, namely: the age (more than 60 years old for women and more than 50 years old for men), the masculine sex, hypertension, diabetes, dyslipidemia, psycho-social or occupational stress, obesity, active or passive smoking, sedentary lifestyle, menopause, cardiovascular inheritance, and chronic renal insufficiency.

We studied the time to admission (time between the onset of chest pain and the patient’s arrival at the cardiac ICU). The general examination data were studied as well as paraclinic data including markers of myocardial ischemia (Troponin I), 18-lead ECG and doppler echocardiography. The therapeutic used during the hospitalization and evolutionary aspects were studied.

### Statistical analysis

Epidemiological, clinical, paraclinical, therapeutic, and evolutionary data were gathered using an exploitation sheets including the various variables collected from patient’s medical records. Then, they were computerized and analyzed using IBM SPSS Statistics 26. The Kolmogorov–Smirnov test was applied to determine the distribution of the data. A *P*-value <0.05 was considered statistically significant. The mean and SD were used for normally distributed variables, and the median and interquartile range were used for variables not normally distributed, while the categorical variables were presented as frequencies and percentages. In view of the retrospective design of this study, the requirement for patient consent was disregarded.

This study was performed in compliance with the standards and principles set forth by the preferred reporting of case series in surgery (PROCESS) guidelines^4^.

## Results

**Figure 1:**
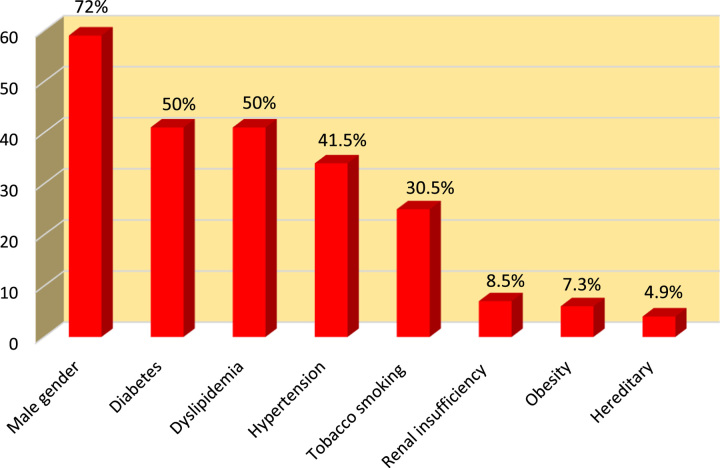
Distribution of patients according to cardiovascular risk factors (*N*=82 ).

During the period of our study, 500 patients were admitted to the cardiological ICU for STEMI, of whom 82 had MI extended to the right ventricle, reflecting a rate of 16.4%. RVI co-existed with inferior MI in 62.2% of cases and in 37.8% of anterior MI, while isolated RVI was seen in only one patient. The mean age in our study was 64±12.3 years with extremes of 44 and 88 years and a higher prevalence between 55 and 70 years, with a male predominance (72%) (sex-ratio=2.5). Diabetes and dyslipidemia are the main cardiovascular risk factors identified in our study, with a prevalence of 50% of cases, arterial hypertension, and smoking are present in 41.5, and 30.5% of cases, respectively, 8.5% had a chronic renal failure, 7.3% of patients were obese, and 4.9% had a history of coronary heart disease (Fig. 1). Chest pain was present in all patients; associated symptoms of nausea and vomiting were present in 42.7% of the patients in our study, sweating in 11% of cases, and with dyspnea in 9.8% patients. Syncope and faintness were reported by 3.6% of patients, 7.3% had hypotension (SBP <90 mmHg). Regarding the consultation time, 29.3% of patients presented to the hospital 12 h after the onset of symptoms. Among our patients, 36.6% of them presented clinical signs of right heart failure and only 2.4% of patients had signs of left heart failure.

The ECG showed an ST elevation in leads DII-DIII-AVF with extension to right ventricle in 62.2% of patients and ST elevation in leads V1 to V3 with extension to right ventricle in 37.8% of cases, while isolated RVMI was seen in only one patient. Complete arrhythmia by atrial fibrillation is found in 4.9% of patients. Atrioventricular block is found in 15.8% of patients. Third-degree atrioventricular block was diagnosed in 8.5% of patients (Table 2).

**Table 2:** Electrocardiographic findings of RVMI.

ECG	Number of cases (%)
ST Elevation in leads V3R-V4R	82 (100%)
ST Elevation in leads DII-DIII-AVF	51 (62.2%)
ST Elevation in leads V1-V3	31 (37.8%)
Sinus bradycardia	12 (14.6%)
Second-degree AV block	2 (2.4%)
Third-degree AV block	7 (8.5%)
Atrial fibrillation	4 (4.9%)
Ventricular tachycardia /	4 (4.9%)
Ventricular fibrillation	2 (2.4%)

Transthoracic echocardiography showed RV systolic dysfunction in 24.39% of cases, while RV dilatation was seen in only 10.9% of patients. In our study, the LVEF ranged from 15 to 67% with a median of 35±12.8%. Segmental wall motion abnormalities were consistent with ECG findings and were more prevalent in the inferior segment it was akinetic in 45.1% in cases followed by the infero-septo-lateral wall in 17.1% of cases and the antero-septo-lateral wall in 37.8% of cases, 7.3% of patients presented with low to moderate pericardial effusion without signs of tamponade, mitral insufficiency was objectified in 35.3% of patients, and tricuspid insufficiency in 9.7% of patients.

Laboratory investigations revealed elevated levels of troponin in all our patients and renal failure in 8.5% of patients.

Among the 82 patients included in the study, 46.3% of patients received thrombolytic treatments, which was successful in 28.04% of cases. All patients received standard treatment with statins, double antiplatelet aggregation, low-molecular-weight heparin. The beta-blockers and the angiotensin-converting enzyme inhibitor were prescribed in 79.2%, and 60.9% of cases, respectively. The use of dobutamine was requested in 7.3% of patients and vascular filling was indicated in 6.09% of patients.

**Figure 2:**
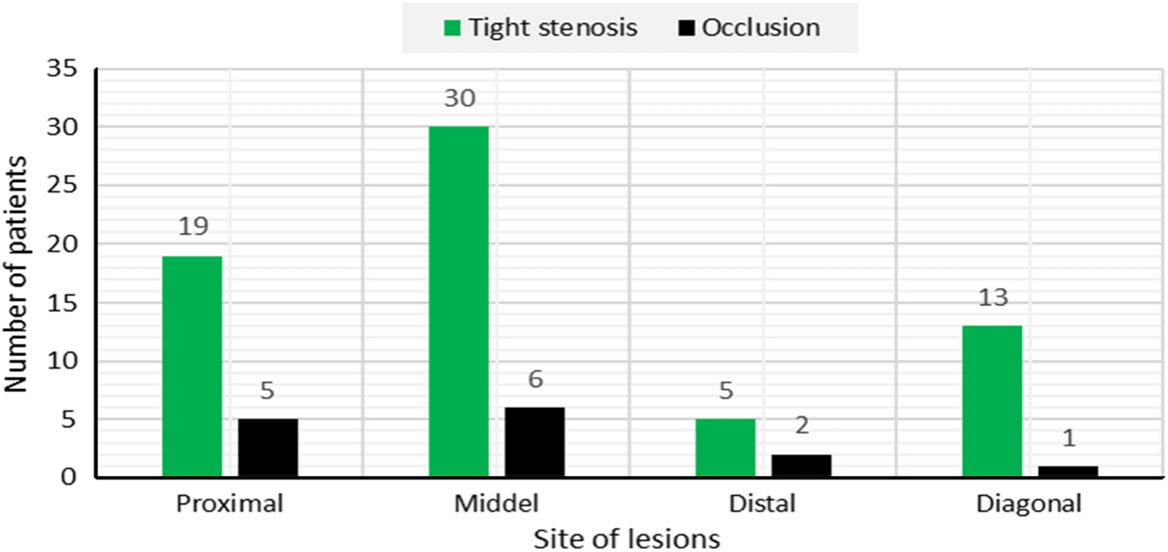
Distribution of tight stenoses and occlusions of the anterior interventricular artery.

**Figure 3:**
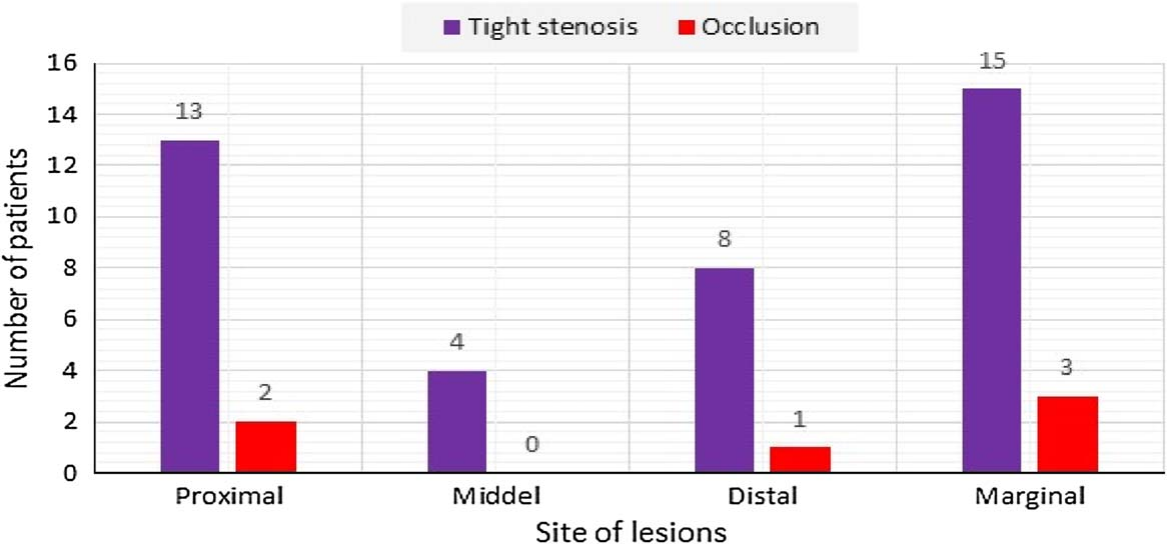
Distribution of tight stenoses and occlusions of the circumflex.

**Figure 4:**
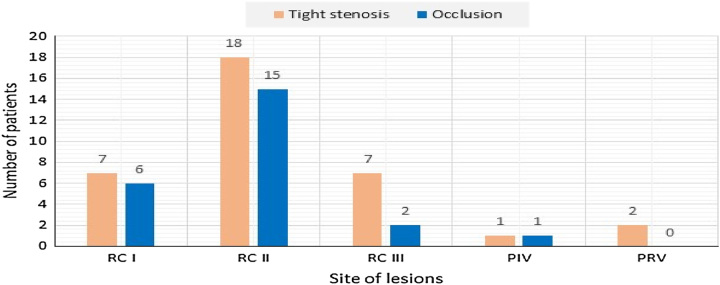
Distribution of tight stenoses and occlusions of the right coronary artery.

Coronary angiography was performed in 89.02% of patients, mainly with a radical approach (82.92%). Right dominance was present in 60.97% of cases. The prevalence of left and balanced dominance were 8.55 and 30.48%, respectively. The distribution of tight stenoses and occlusions of the anterior interventricular artery, circumflex, and right coronary arteries are shown respectively in (Figs 2, 3, and 4). The culprit lesion of the inferior wall MI was the RCA in 59.75% of patients and the left circumflex artery in 15.85% of patients. The culprit lesion of the anterior wall MI was the anterior interventricular artery in 58.53% of patients and the circumflex artery in 6.09% of patients. Coronary angioplasty by active stents was performed in 76.82% of cases, the length of the stents varies between 12 mm and 36 mm with a diameter between 2.25 mm and 3.5 mm. A successful angioplasty was defined as the restoration of TIMI III flow in 73.2% of cases. For the other patients, we completed a myocardial viability testing in 9.75% of patients to make a therapeutic decision. We opted for medical treatment for 3.65% of patients and coronary artery bypass grafting for 3.65% of patients. Temporary pacing was required in 2.4% of cases.

In-hospital complications were observed in 52.28% of our study, these complications include hypotension in 7.31% of cases, cardiogenic shock in 4.87% of cases, conduction disorders were seen in 15.8% of cases, 8.5% of patients presented with third-degree atrioventricular block, 7.3% each with first-degree and second-degree atrioventricular block, bradyarrhythmia without hemodynamic instability were noted in 14.6%, ventricular fibrillation was noted in 2.4%, ventricular tachycardia in 4.9%, and 2.4% of patients died.

## Discussion

MI can affect any myocardial territory; however, isolated RVI is very rare^2,5^. Nonetheless, the prognosis of inferior MI is better in both the short and the long term than anterior MI^6^. Acute RVMI is frequently associated with inferior wall MI (30–50%) and less frequently with anterior wall MI^7–9^, this association worsens the prognosis with a mortality of about 30% compared to that of isolated left ventricular infarctions (6%)^7^. The prevalence of RVI in the present study was 16.4%.

Unlike left ventricular infarction, which has been extensively described and studied since antiquity, RVI has been described as a distinct clinical entity since 1974 (Cohn, 1974). In our series, RVMI co-existed with inferior MI in 62.2% of cases and in 37.8% of anterior MI, while isolated RVMI was seen in only one patient, which is similar to others studies^2,5,6^.

In our study, the average age was 64±12.3 years, which is superior to what is found by Fennira *et al*.^2^ Male sex predominance was also found (72%), thing that is similar in other studies^2,7^. The majority of our patients had more cardiovascular risk factors. High blood pressure is a real public health problem and is one of the major important risk factors for coronary heart disease^10^, it is found in 41.5% of patients in our series. Smoking is the main cardiovascular risk factor. In our study, it is found in 30.5% of cases, this rate is similar to that found by Ngaïdé *et al*. (30%)^11^. Patients with diabetes have 2–4 times increased risk of cardiovascular morbidity and mortality than individuals without diabetes as demonstrated in the United Kingdom prospective diabetes study^12^. In our study, it is found in 50% of patients. It is clearly shown, in the FRAMINGHAM research that there is a solid link between elevated cholesterol (especially LDL-C) and cardiovascular disease^13^. In our study, dyslipidemia was present in 50% of cases, which is superior than that found by Ngaide *et al*. (40%)^11^.

The clinical trial of hypotension, clear lung fields, and elevated jugular venous pressure has been traditionally considered as a maker of RVI in patients with acute inferior wall MI. However, this triad has high specificity (96%) but very low sensitivity (25%)^14^.

ECG is an important tool for the diagnosis as signs and symptoms are not very specific. The standard 12‑lead ECG gives a better picture of the left side of the heart compared to the right. Right-sided precordial lead ECG is crucial for the diagnosis . RVI is suspected when there is ST segment elevation in lead V4R correlates closely with occlusion of the proximal right coronary artery, and has 88 sensitivity and 78% specificity for concurrent RVMI in patients with IWMI^15^. Erhardt and Sjogren^16^ used a more complicated single lead V4R and found it to be positive in 25 out of 92 cases, all of whom had RVI on autopsy. Erhardt and Sjogren found out and pointed out that the pathogenesis of changes in right precordial leads may be a result of either transmural RV damage or infarction of the posterior interventricular septum, reflected through the necrosis and electrically silent RV myocardium.

Transthoracic doppler echocardiography has an important role in the diagnosis of RVI, findings are also unspecific and may include dilated right cardiac chambers, RV wall motion abnormalities mostly akinesia of the posteroinferior wall, paradoxical movement of interventricular septum, RV systolic dysfunction, increased systolic pulmonary artery pressure and dilated inferior vena cava^17^. In our series, RV systolic dysfunction in 24.39% of cases, while RV dilatation was seen in only 10.9% of patients.

According to many larges studies, MRI is the gold standard for the exploration of RV dysfunction^18^.

Complications are frequent in the acute phase dominated by signs of right heart failure, cardiovascular collapse and atrioventricular conduction disorders^19^. The major cause of death in patients with acute RVMI is cardiogenic shock. Garg *et a1*.^20^ found RHF and hypotension in 21.2% of their patients who suffered from RVI. The present study is in consistent with the above mentioned studies. Complete atrioventricular block and bundle-branch block are the most frequent cardiac conduction disorders associated with RV infarctio. In our series, 15.8% of patients presented atrioventricular blocks. Our findings are comparable with those reported by Braat *et al*.^21^ and Garg *et al*.^20^ In higher incidence of second or third-degree heart blocks in association with RVI is probably due to the involvement of the region of atrioventricular node which is supplied by right coronary artery.

Early diagnosis in this situation is important to ensure not only appropriate treatment but also to ensure that potentially dangerous therapies, such as vasodilators, nitrates, and morphine.

Effective fluid resuscitation aiming to restore the preload, and subsequently maintain adequate cardiac output, along with percutaneous or pharmacological revascularization is first-line therapy of acute RVMI, it tends to decrease the high intrahospital mortality, improve prognosis, and reduce complications. Nevertheless, successful thrombolysis has an important impact on the recovery of right ventricle function and overall survival^22^. 46.3% of our patients received thrombolytic therapy, which was successful in 28.04% of cases. In addition to the reperfusion, the use of dobutamine was requested in 7.3% of patients and vascular filling was indicated in 6.09% of patients. Coronary angiography was performed in 89.02% of patients and 76.82% patients received Percutaneous Coronary Intervention of the culprit artery following coronary angiography.

Angiographically, RVI complicating acute inferior MI is generally due to occlusion of the right coronary artery. Occlusion of the circumflex artery can sometimes accompany RVI when it is dominant. Occlusion of the anterior interventricular artery is often accompanied by a small RVI that goes unnoticed and has no clinical consequences^23^. In our series, inferior MI was caused by right coronary artery in 59.75% of patients and circumflex artery in 15.85% of patients. Anterior MI was caused by anterior interventricular artery in 58.53% of patients and circumflex artery in 6.09% of patients.

Clinical implications must be taken into consideration to come up with acceptable solutions for this significant challenge. It is therefore very important to early recognize the RV involvement in a patient presenting with acute MI, not only for prognosis, but also to choose the specific therapy, including aggressive primary PCI, with particular attention to RV branch revascularization, all in order to avoid any unwanted detrimental complications associated with this diagnosis.

Our research has some limitations. Present study involved only 82 patients and there was no validation or proper documentation of true RVI by means of nuclear scan imaging and contrast ventriculography. We relied on previous studies, which had already validated ECG as a reliable indicator of MI.

Future research with prospective enrollment and longer follow-up is needed. Notably, 18-lead ECG is recommended for all patients with STEMI. Therefore, these patients need to be adequately supported earlier in the process of their treatment and follow-ups with reduced time periods to avoid any complications.

## Conclusion

RVI is a rare entity in acute coronary syndromes. The right ventricle is distinguished by its anatomo-physiological peculiarities responsible for its physiopathological singularity explaining the advent of various complications. The diagnosis of RVMI can be challenging; the 12 lead ECGs with supplemental right precordial recordings remain the principal diagnostic tool. Early diagnosis, prompt treatment, and appropriate of acute RVMI are the keys to improve prognosis, and reduce complications.

## Ethical approval

This study does not require a formal ethical committee approval. Access to patient data was authorized by the director of Mohammed VI hospital university and approved by the head of the department, taking into account the retrospective design of this study.

## Consent

Informed consent was waived due to the study’s retrospective design, based on our institutional policies for deidentified case series.

## Sources of funding

No funding was received for this work.

## Conflicts of interest disclosures

The authors declare no relevant competing interests related to the content of this article.

## Research registration unique identifying number (UIN)

Researchregistry7824.

## Guarantor

Dr Bouhadoune Youssra.

## Data availability statement

None.

## Provenance and peer review

Not commissioned, externally peer-reviewed.
